# Constrained Bayesian optimization for automatic chemical design using variational autoencoders†
†Electronic supplementary information (ESI) available: Additional experimental results validating the algorithm configuration on the toy Branin-Hoo function. See DOI: 10.1039/c9sc04026a


**DOI:** 10.1039/c9sc04026a

**Published:** 2019-11-18

**Authors:** Ryan-Rhys Griffiths, José Miguel Hernández-Lobato

**Affiliations:** a Cavendish Laboratory , Department of Physics , University of Cambridge , UK . Email: rrg27@cam.ac.uk; b Department of Engineering , University of Cambridge , UK . Email: jmh233@cam.ac.uk; c Alan Turing Institute , London , UK; d Microsoft Research , Cambridge , UK

## Abstract

Automatic Chemical Design is a framework for generating novel molecules with optimized properties. The original scheme, featuring Bayesian optimization over the latent space of a variational autoencoder, suffers from the pathology that it tends to produce invalid molecular structures. First, we demonstrate empirically that this pathology arises when the Bayesian optimization scheme queries latent space points far away from the data on which the variational autoencoder has been trained. Secondly, by reformulating the search procedure as a constrained Bayesian optimization problem, we show that the effects of this pathology can be mitigated, yielding marked improvements in the validity of the generated molecules. We posit that constrained Bayesian optimization is a good approach for solving this kind of training set mismatch in many generative tasks involving Bayesian optimization over the latent space of a variational autoencoder.

## Abstract 2

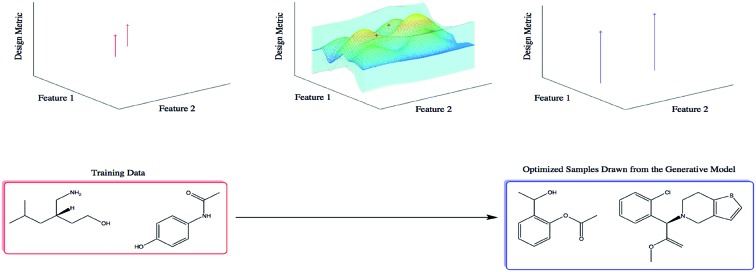
Automatic Chemical Design is a framework for generating novel molecules with optimized properties.

## Introduction

Machine learning in chemical design has shown promise along a number of fronts. In quantitative structure activity relationship (QSAR) modelling, deep learning models have achieved state-of-the-art results in molecular property prediction^1^–^8^ as well as property uncertainty quantification.^9^–^12^ Progress is also being made in the interpretability and explainability of machine learning solutions to chemical design, a subfield concerned with extracting chemical insight from learned models.^13^ The focus of this paper however, will be on molecule generation, leveraging machine learning to propose novel molecules that optimize a target objective.

One existing approach for finding molecules that maximize an application-specific metric involves searching a large library of compounds, either physically or virtually.^14^,^15^ This has the disadvantage that the search is not open-ended; if the molecule is not contained in the library, the search won't find it.

A second method involves the use of genetic algorithms. In this approach, a known molecule acts as a seed and a local search is performed over a discrete space of molecules. Although these methods have enjoyed success in producing biologically active compounds, an approach featuring a search over an open-ended, continuous space would be beneficial. The use of geometrical cues such as gradients to guide the search in continuous space in conjunction with advances in Bayesian optimization methodologies^16^,^17^ could accelerate both drug^14^,^18^ and materials^19^,^20^ discovery by functioning as a high-throughput virtual screen of unpromising candidates.

Recently, Gómez-Bombarelli *et al.*^21^ presented Automatic Chemical Design, a variational autoencoder (VAE) architecture capable of encoding continuous representations of molecules. In continuous latent space, gradient-based optimization is leveraged to find molecules that maximize a design metric.

Although a strong proof of concept, Automatic Chemical Design possesses a deficiency in so far as it fails to generate a high proportion of valid molecular structures. The authors hypothesize^21^ that molecules selected by Bayesian optimization lie in “dead regions” of the latent space far away from any data that the VAE has seen in training, yielding invalid structures when decoded.

The principle contribution of this paper is to present an approach based on constrained Bayesian optimization that generates a high proportion of valid sequences, thus solving the training set mismatch problem for VAE-based Bayesian optimization schemes.

## Methods

### SMILES representation

**Fig. 1:**
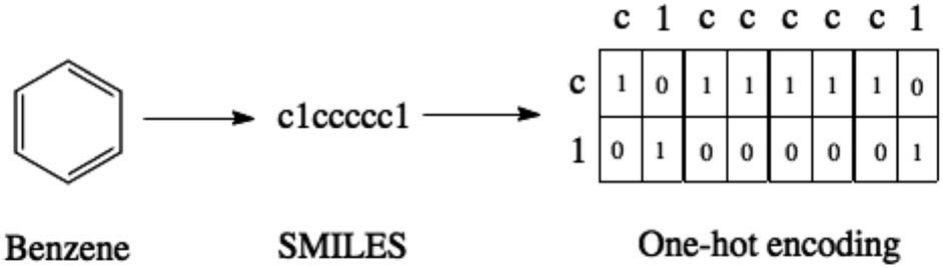
The SMILES representation and one-hot encoding for benzene. For purposes of illustration, only the characters present in benzene are shown in the one-hot encoding. In practice there is a column for each character in the SMILES alphabet.

SMILES strings^22^ are a means of representing molecules as a character sequence. This text-based format facilitates the use of tools from natural language processing for applications such as chemical reaction prediction^23^–^28^ and chemical reaction classification.^29^ To make the SMILES representation compatible with the VAE architecture, the SMILES strings are in turn converted to one-hot vectors indicating the presence or absence of a particular character within a sequence as illustrated in Fig. 1.

### Variational autoencoders

Variational autoencoders^30^,^31^ allow us to map molecules **m** to and from continuous values **z** in a latent space. The encoding **z** is interpreted as a latent variable in a probabilistic generative model over which there is a prior distribution *p*(**z**). The probabilistic decoder is defined by the likelihood function *p*_*θ*_(**m**|**z**). The posterior distribution *p*_*θ*_(**z**|**m**) is interpreted as the probabilistic encoder. The parameters of the likelihood *p*_*θ*_(**m**|**z**) as well as the parameters of the approximate posterior distribution *q*_*φ*_(**z**|**m**) are learned by maximizing the evidence lower bound (ELBO)
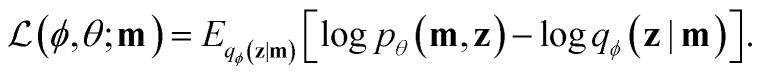


**Fig. 2:**
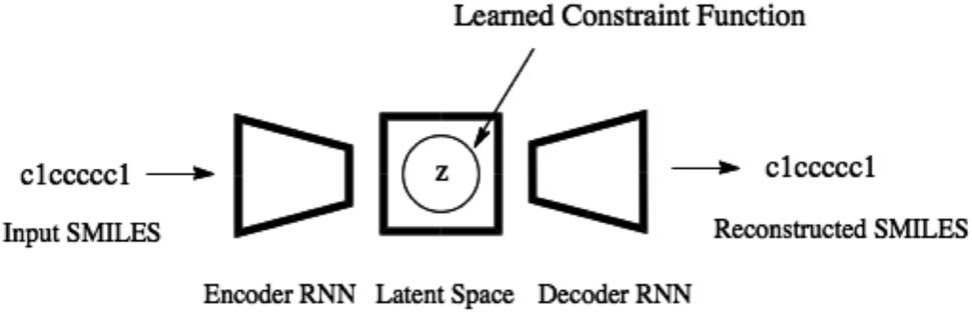
The SMILES variational autoencoder with the learned constraint function illustrated by a circular feasible region in the latent space.

Variational autoencoders have been coupled with recurrent neural networks by ref. 32 to encode sentences into a continuous latent space. This approach is followed for the SMILES format both by ref. 21 and here. The SMILES variational autoencoder, together with our constraint function, is shown in Fig. 2.

### The origin of dead regions in the latent space

The approach introduced in this paper aims to solve the problem of dead regions in the latent space of the VAE. It is first however, important to understand the origin of these dead zones. Three ways in which a dead zone can arise are:

(1) Sampling locations that are very unlikely under the prior. This was noted in the original paper on variational autoencoders^30^ where sampling was adjusted through the inverse conditional distribution function of a Gaussian.

(2) A latent space dimensionality that is artificially high will yield dead zones in the manifold learned during training.^33^ This has been demonstrated to be the case empirically in ref. 34.

(3) Inhomogenous training data; undersampled regions of the data space are liable to yield gaps in the latent space.

**Fig. 3:**
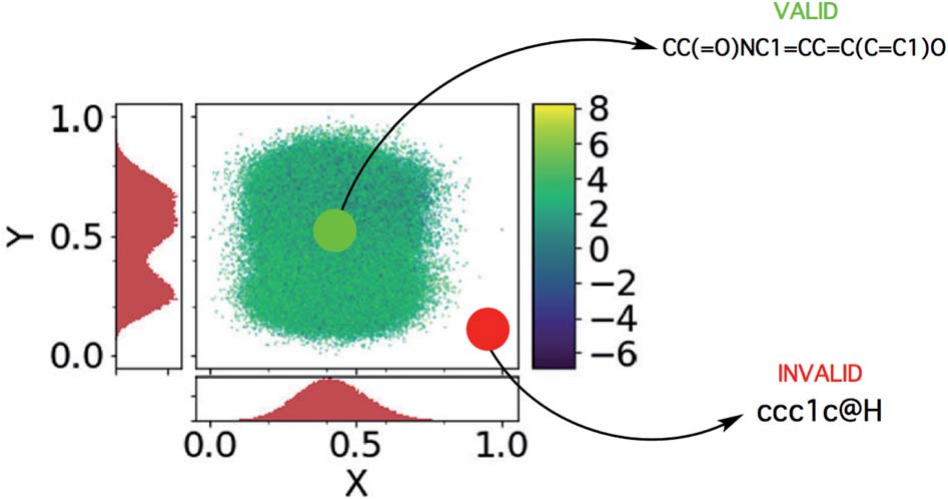
The dead zones in the latent space, adapted from ref. 21. The *x* and *y* axes are the principle components computed by PCA. The colour bar gives the log *P* value of the encoded latent points and the histograms show the coordinate-projected density of the latent points. One may observe that the encoded molecules are not distributed uniformly across the box constituting the bounds of the latent space.

A schematic illustrating sampling from a dead zone, and the associated effect it has on the generated SMILES strings, is given in Fig. 3. In our case, the Bayesian optimization scheme is decoupled from the VAE and hence has no knowledge of the location of the learned manifold. In many instances the explorative behaviour in the acquisition phase of Bayesian optimization will drive the selection of invalid points lying far away from the learned manifold.

### Objective functions for Bayesian optimization of molecules

Bayesian optimization is performed here in the latent space of the variational autoencoder in order to find molecules that score highly under a specified objective function. We assess molecular quality on the following objectives:*J*_comp_^log^ ^*P*^(**z**) = log *P*(**z**) – SA(**z**) – ring-penalty(**z**),*J*_comp_^QED^(**z**) = QED(**z**) – SA(**z**) – ring-penalty(**z**),*J*^QED^(**z**) = QED(**z**).**z** denotes a molecule's latent representation, log *P*(**z**) is the water–octanol partition coefficient, QED(**z**) is the quantitative estimate of drug-likeness^35^ and SA(**z**) is the synthetic accessibility.^36^ The ring penalty term is as featured in ref. 21. The “comp” subscript is designed to indicate that the objective function is a composite of standalone metrics.

It is important to note, that the first objective, a common metric of comparison in this area, is misspecified as has been pointed out by ref. 37. From a chemical standpoint it is undesirable to maximize the log *P* score as is being done here. Rather it is preferable to optimize log *P* to be in a range that is in accordance with the Lipinski rule of five.^38^ We use the penalized log *P* objective here because regardless of its relevance for chemistry, it serves as a point of comparison against other methods.

### Constrained Bayesian optimization of molecules

We now describe our extension to the Bayesian optimization procedure followed by ref. 21. Expressed formally, the constrained optimization problem is
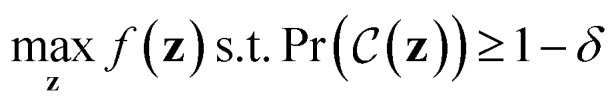
where *f*(**z**) is a black-box objective function, 
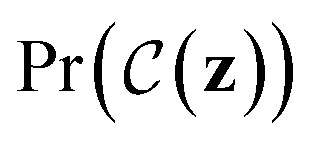
 denotes the probability that a Boolean constraint 
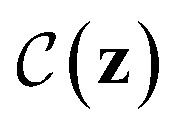
 is satisfied and 1 – *δ* is some user-specified minimum confidence that the constraint is satisfied.^39^ The constraint is that a latent point must decode successfully a large fraction of the times decoding is attempted. The specific fractions used are provided in the results section. The black-box objective function is noisy because a single latent point may decode to multiple molecules when the model makes a mistake, obtaining different values under the objective. In practice, *f*(**z**) is one of the objectives described in Section 2.3.

### Expected improvement with constraints (EIC)

EIC may be thought of as expected improvement (EI),EI(**z**) = *E*_*f*(**z**)_[max(0,*f*(**z**) – *η*)],that offers improvement only when a set of constraints are satisfied:^40^
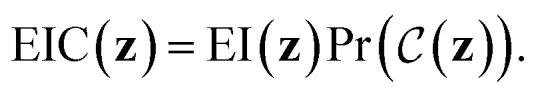


The incumbent solution *η* in EI(**z**), may be set in an analogous way to vanilla expected improvement^41^ as either:

(1) The best observation in which all constraints are observed to be satisfied.

(2) The minimum of the posterior mean such that all constraints are satisfied.

The latter approach is adopted for the experiments performed in this paper. If at the stage in the Bayesian optimization procedure where a feasible point has yet to be located, the form of acquisition function used is that defined by ref. 41.
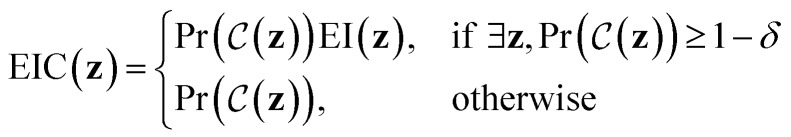
with the intuition being that if the probabilistic constraint is violated everywhere, the acquisition function selects the point having the highest probability of lying within the feasible region. The algorithm ignores the objective until it has located the feasible region.

### Related work

The literature concerning generative models of molecules has exploded since the first work on the topic.^21^ Current methods feature molecular representations such as SMILES^42^–^54^ and graphs^55^–^72^ and employ reinforcement learning^73^–^83^ as well as generative adversarial networks^84^ for the generative process. These methods are well-summarized by a number of recent review articles.^85^–^89^ In terms of VAE-based approaches, two popular approaches for incorporating property information into the generative process are Bayesian optimization and conditional variational autoencoders (CVAEs).^90^ When generating molecules using CVAEs, the target data *y* is embedded into the latent space and conditional sampling is performed^47^,^91^ in place of a directed search *via* Bayesian optimization. In this work we focus solely on VAE-based Bayesian optimization schemes for molecule generation and so we do not benchmark model performance against the aforementioned methods. Principally, we are concerned with highlighting the issue of training set mismatch in VAE-based Bayesian optimizations schemes and demonstrating the superior performance of a constrained Bayesian optimization approach.

## Results and discussion

### Experiment I

#### Drug design

In this section we conduct an empirical test of the hypothesis from ref. 21 that the decoder's lack of efficiency is due to data point collection in “dead regions” of the latent space far from the data on which the VAE was trained. We use this information to construct a binary classification Bayesian Neural Network (BNN) to serve as a constraint function that outputs the probability of a latent point being valid, the details of which will be discussed in the section on labelling criteria. The BNN implementation is adapted from the MNIST digit classification network of ref. 92 and is trained using black-box alpha divergence minimization. Secondly, we compare the performance of our constrained Bayesian optimization implementation against the original model (baseline) in terms of the numbers of valid, realistic and drug-like molecules generated. We introduce the concept of a realistic molecule *i.e.* one that has a SMILES length greater than 5 as a heuristic to gauge whether the decoder has been successful or not. Our definition of drug-like is that a molecule must pass 8 sets of structural alerts or functional group filters from the ChEMBL database.^93^ Thirdly, we compare the quality of the molecules produced by constrained Bayesian optimization with those of the baseline model. The code for all experiments has been made publicly available at ; https://github.com/Ryan-Rhys/Constrained-Bayesian-Optimisation-for-Automatic-Chemical-Design.

#### Implementation

The implementation details of the encoder-decoder network as well as the sparse GP for modelling the objective remain unchanged from ref. 21. For the constrained Bayesian optimization algorithm, the BNN is constructed with 2 hidden layers each 100 units wide with ReLU activation functions and a logistic output. Minibatch size is set to 1000 and the network is trained for 5 epochs with a learning rate of 0.0005. 20 iterations of parallel Bayesian optimization are performed using the Kriging-Believer algorithm^94^ in all cases. Data is collected in batch sizes of 50. The same training set as ref. 21 is used, namely 249, 456 drug-like molecules drawn at random from the ZINC database.^95^

#### Diagnostic experiments and labelling criteria

**Fig. 4:**
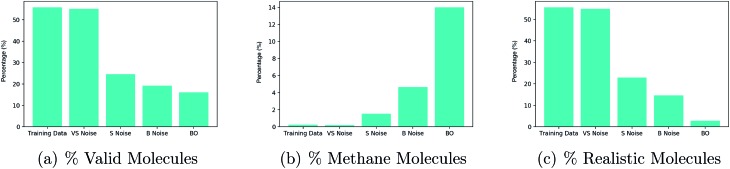
Experiments on 5 disjoint sets comprising 50 latent points each. Very small (VS) noise are training data latent points with approximately 1% noise added to their values, small (S) noise have 10% noise added to their values and big (B) noise have 50% noise added to their values. All latent points underwent 500 decode attempts and the results are averaged over the 50 points in each set. The percentage of decodings to: (a) valid molecules (b) methane molecule, (c) realistic molecules.

These experiments were designed to test the hypothesis that points collected by Bayesian optimization lie far away from the training data in latent space. In doing so, they also serve as labelling criteria for the data collected to train the BNN acting as the constraint function. The resulting observations are summarized in Fig. 4.

There is a noticeable decrease in the percentage of valid molecules decoded as one moves further away from the training data in latent space. Points collected by Bayesian optimization do the worst in terms of the percentage of valid decodings. This would suggest that these points lie farthest from the training data. The decoder over-generates methane molecules when far away from the data. One hypothesis for why this is the case is that methane is represented as ‘C’ in the SMILES syntax and is by far the most common character. Hence far away from the training data, combinations such as ‘C’ followed by a stop character may have high probability under the distribution over sequences learned by the decoder.

Given that methane has far too low a molecular weight to be a suitable drug candidate, a third plot in Fig. 3(c), shows the percentage of decoded molecules such that the molecules are both valid and have a tangible molecular weight. The definition of a tangible molecular weight was interpreted somewhat arbitrarily as a SMILES length of 5 or greater. Henceforth, molecules that are both valid and have a SMILES length greater than 5 will be referred to as realistic. This definition serves the purpose of determining whether the decoder has been successful or not.

As a result of these diagnostic experiments, it was decided that the criteria for labelling latent points to initialize the binary classification neural network for the constraint would be the following: if the latent point decodes into realistic molecules in more than 20% of decode attempts, it should be classified as realistic and non-realistic otherwise.

#### Molecular validity

The BNN for the constraint was initialized with 117 440 positive class points and 117 440 negative class points. The positive points were obtained by running the training data through the decoder assigning them positive labels if they satisfied the criteria outlined in the previous section. The negative class points were collected by decoding points sampled uniformly at random across the 56 latent dimensions of the design space. Each latent point undergoes 100 decode attempts and the most probable SMILES string is retained. *J*_comp_^log^ ^*P*^(**z**) is the choice of objective function. The raw validity percentages for constrained and unconstrained Bayesian optimization are given in Table 1.

**Table 1:** Percentage of valid molecules produced

Run	Baseline	Constrained
1	29%	94%
2	51%	97%
3	12%	90%
4	37%	93%
5	49%	86%

**Fig. 5:**
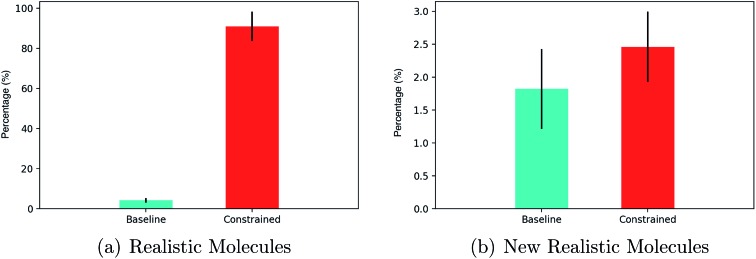
(a) The percentage of latent points decoded to realistic molecules. (b) The percentage of latent points decoded to unique, novel realistic molecules. The results are from 20 iterations of Bayesian optimization with batches of 50 data points collected at each iteration (1000 latent points decoded in total). The standard error is given for 5 separate train/test set splits of 90/10.

In terms of realistic molecules, the relative performance of constrained Bayesian optimization and unconstrained Bayesian optimization (baseline)^21^ is compared in Fig. 5(a).

The results show that greater than 80% of the latent points decoded by constrained Bayesian optimization produce realistic molecules compared to less than 5% for unconstrained Bayesian optimization. One must account however, for the fact that the constrained approach may be decoding multiple instances of the same novel molecules. Constrained and unconstrained Bayesian optimization are compared on the metric of the percentage of unique novel molecules produced in Fig. 5(b).

One may observe that constrained Bayesian optimization outperforms unconstrained Bayesian optimization in terms of the generation of unique molecules, but not by a large margin. A manual inspection of the SMILES strings collected by the unconstrained optimization approach showed that there were many strings with lengths marginally larger than the cutoff point, which is suggestive of partially decoded molecules. We run a further test of drug-likeness for the unique novel molecules generated by both methods consisting of passing a number of functional group filters consisting of 8 sets of structural alerts from the ChEMBL database. The alerts consisted of the Pan Assay Interference Compounds (PAINS)^96^ alert set for nuisance compounds that elude usual reactivity, the NIH MLSMR alert set for excluded functionality filters, the Inpharmatica alert set for unwanted fragments, the Dundee alert set,^97^ the BMS alert set,^98^ the Pfizer Lint procedure alert set^99^ and the Glaxo Wellcome alert set.^100^ An additional screen dictating that molecules should have a molecular weight between 150–500 daltons was also included. The results are given in Table 2.

**Table 2:** Percentage of novel generated molecules passing ChemBL structural alerts

Baseline	Constrained
6.6%	35.7%

In the next section we compare the quality of the novel molecules produced as judged by the scores from the black-box objective function.

#### Molecular quality

**Fig. 6:**
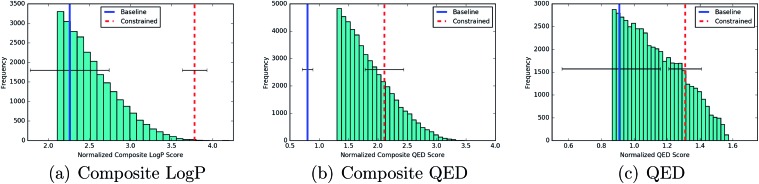
The best scores for new molecules generated from the baseline model (blue) and the model with constrained Bayesian optimization (red). The vertical lines show the best scores averaged over 5 separate train/test splits of 90/10. For reference, the histograms are presented against the backdrop of the top 10% of the training data in the case of composite log *P* and QED, and the top 20% of the training data in the case of composite QED.

The results of Fig. 6 indicate that constrained Bayesian optimization is able to generate higher quality molecules relative to unconstrained Bayesian optimization across the three drug-likeness metrics introduced in Section 2.3. Over the 5 independent runs, the constrained optimization procedure in every run produced new molecules ranked in the 100th percentile of the distribution over training set scores for the *J*_comp_^log^ ^*P*^(**z**) objective and over the 90th percentile for the remaining objectives. Table 3 gives the percentile that the averaged score of the new molecules found by each process occupies in the distribution over training set scores. The *J*_comp_^log^ ^*P*^(**z**) objective is included as a metric for the generative performance of the models. It has been previously noted that it should not be beneficial for the purposes of drug design.

**Table 3:** Percentile of the averaged new molecule score relative to the training data. The results of 5 separate train/test set splits of 90/10 are provided

Objective	Baseline	Constrained
log *P* composite	36 ± 14	92 ± 4
QED composite	14 ± 3	72 ± 10
QED	11 ± 2	79 ± 4

**Fig. 7:**
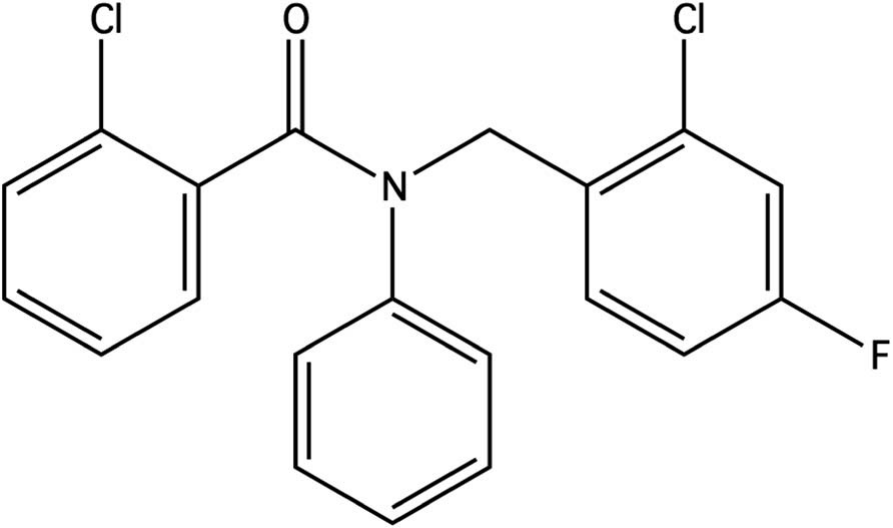
The best molecule obtained by constrained Bayesian optimization as judged by the penalised log *P* objective function score.

For the penalised log *P* objective function, scores for each run are presented in Table 4. The best score obtained from our constrained Bayesian optimization approach is compared against the scores reported by other methods in Table 5. The best molecule under the penalised log *P* objective obtained from our method is depicted in Fig. 7.

**Table 4:** Penalised log *P* objective scores with the best score obtained highlighted in bold

Run	Baseline	Constrained
1	2.02	**4.01**
2	2.81	3.86
3	1.45	3.62
4	2.56	3.82
5	2.47	3.63

**Table 5:** Comparison of penalised log *P* objective function scores against other models. Note that the results are taken from the original works and as such don't constitute a direct performance comparison due to different run configurations

Grammar VAE^59^	Constrained BO VAE	SD-VAE^60^	JT-VAE^57^
2.94	4.01	4.04	5.30

### Experiment II

#### Combining molecule generation and property prediction

In order to show that the constrained Bayesian optimization approach is extensible beyond the realm of drug design, we trained the model on data from the Harvard Clean Energy Project^19^,^20^ to generate molecules optimized for power conversion efficiency (PCE). In the absence of ground truth values for the PCE of the novel molecules generated, we use the output of a neural network trained to predict PCE as a surrogate. As such, the predictive accuracy of the property prediction model will be a bottleneck for the quality of the generated molecules.

#### Implementation

A Bayesian neural network with 2 hidden layers and 50 ReLU units per layer was trained to predict the PCE of 200 000 molecules drawn at random from the Harvard Clean Energy Project dataset using 512 bit Morgan circular fingerprints^101^ as input features with bond radius of 2 computed using RDKit.^102^ While a larger radius may be appropriate for the prediction of PCE in order to represent conjugation, we are only interested in showing how a property predictor might be incorporated into the automatic chemical design framework and not in optimizing that predictor. The network was trained for 25 epochs with the ADAM optimizer^103^ using black box alpha divergence minimization with an alpha parameter of 5, a learning rate of 0.01, and a batch size of 500. The RMSE on the training set of 200 000 molecules is 0.681 and the RMSE on the test set of 25 000 molecules is 0.999.

#### PCE scores

**Fig. 8:**
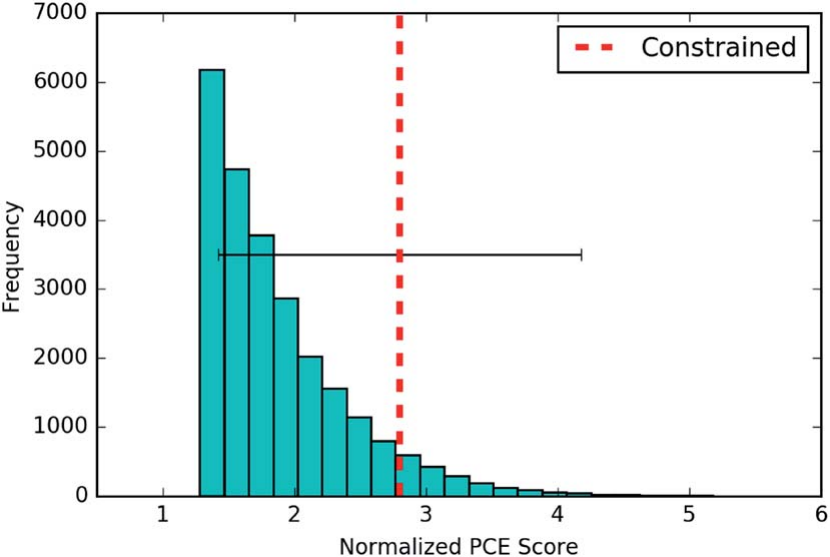
The best scores for novel molecules generated by the constrained Bayesian optimization model optimizing for PCE. The results are averaged over 3 separate runs with train/test splits of 90/10. The PCE score is normalized to zero mean and unit variance by the empirical mean and variance of the training set.

The results are given in Fig. 8. The averaged score of the new molecules generated lies above the 90th percentile in the distribution over training set scores. Given that the objective function in this instance was learned using a neural network, advances in predicting chemical properties from data^104^,^105^ are liable to yield concomitant improvements in the optimized molecules generated through this approach.

## Concluding remarks

The reformulation of the search procedure in the Automatic Chemical Design model as a constrained Bayesian optimization problem has led to concrete improvements on two fronts:

(1) Validity – the number of valid molecules produced by the constrained optimization procedure offers a marked improvement over the original model.

(2) Quality – for five independent train/test splits, the scores of the best molecules generated by the constrained optimization procedure consistently ranked above the 90th percentile of the distribution over training set scores for all objectives considered.

These improvements provide strong evidence that constrained Bayesian optimization is a good solution method for the training set mismatch pathology present in the unconstrained approach for molecule generation. More generally, we foresee that constrained Bayesian optimization is a workable solution to the training set mismatch problem in any VAE-based Bayesian optimization scheme. Our code is made publicly available at https://github.com/Ryan-Rhys/Constrained-Bayesian-Optimisation-for-Automatic-Chemical-Design. Further work could feature improvements to the constraint scheme^106^–^111^ as well as extensions to model heteroscedastic noise.^112^

In terms of objectives for molecule generation, recent work by^44^,^89^,^91^,^113^,^114^ has featured a more targeted search for novel compounds. This represents a move towards more industrially-relevant objective functions for Bayesian optimization which should ultimately replace the chemically misspecified objectives, such as the penalized log *P* score, identified both here and in ref. 37. In addition, efforts at benchmarking generative models of molecules^115^,^116^ should also serve to advance the field. Finally, in terms of improving parallel Bayesian optimization procedures in molecule generation applications one point of consideration is the relative batch size of collected points compared to the dataset size used to initialize the surrogate model. We suspect that in order to gain benefit from sequential sampling the batch size should be on the same order of magnitude as the size of the initialization set as this will induce the uncertainty estimates of the updated surrogate model to change in a tangible manner.

## Conflicts of interest

There are no conflicts to declare.

## Supplementary Material

Supplementary information
