# Supplementary material for: Constrained Bayesian optimization for automatic chemical design using variational autoencoders
Source: Chem Sci. 2019 Nov 18;11(2):577–86. doi: 10.1039/c9sc04026a (PMC7067240; doi:10.1039/c9sc04026a)
Supplement: Supplementary file 1 [file SC-011-C9SC04026A-s001.pdf]

Electronic Supplementary Material (ESI) for Chemical Science.
This journal is © The Royal Society of Chemistry 2019

Supporting Information

Ryan-Rhys Griffiths*,† and José Miguel Hernández-Lobato*,‡,¶,§

†*Cavendish Laboratory, Department of Physics, University of Cambridge*

‡*Department of Engineering, University of Cambridge*

¶*Alan Turing Institute, London, United Kingdom*

§*Microsoft Research, Cambridge, United Kingdom*

E-mail: rrg27@cam.ac.uk; jmh233@cam.ac.uk

Toy Experiment: The Branin-Hoo Function

The Branin-Hoo function is a toy problem on which to test the functionality of the algorithmic implementation for constrained Bayesian optimization. The particular variant of the Branin-Hoo optimization of interest here is the constrained formulation of the problem as featured in.[1] This Branin-Hoo function has three global minima at the coordinates $(-\pi, 12.275), (\pi, 2.275)$ and $(9.42478, 2.475)$. In order to formulate the problem as a constrained optimization problem, a disk constraint on the region of feasible solutions is introduced. In contrast to the formulation of the problem in,[1] the disk constraint is coupled in this scenario in the sense that the objective and the constraint will be evaluated jointly at each iteration of Bayesian optimization. In addition, the observations of the black-box objective function will be assumed to be noise-free. The minima of the Branin-Hoo function as well as the disk constraint are illustrated in Figure 1.

The disk constraint eliminates the upper-left and lower-right solutions, leaving a unique global minimum at $(\pi, 2.275)$. Given that our implementation of constrained Bayesian optimization relies on the use of a sparse GP as the underlying statistical model of the black-box

objective and as such is designed for scale as opposed to performance, the results will not be compared directly against those of[1] who use an exact GP to model the objective. It will be sufficient to compare the performance of the algorithm against random sampling. Both the sequential Bayesian optimization algorithm and the parallel implementation using the Kriging-Believer algorithm are tested.

Implementation

A Sparse GP featuring the FITC approximation, based on the implementation of[2] is used to model the black-box objective function. The kernel choice is exponentiated quadratic with ARD lengthscales. The number of inducing points M was chosen to be 20 in the case of sequential Bayesian optimization, and 5 in the case of parallel Bayesian optimization using the Kriging-Believer algorithm. The sparse GP is trained for 400 epochs using Adam[3] with the default parameters and a learning rate of 0.005. The minibatch size is chosen to be 5. The jitter is chosen to be 0.00001. A Bayesian Neural Network (BNN), adapted from the MNIST digit classification network of[4] is trained using black-box alpha divergence minimization to model the constraint.

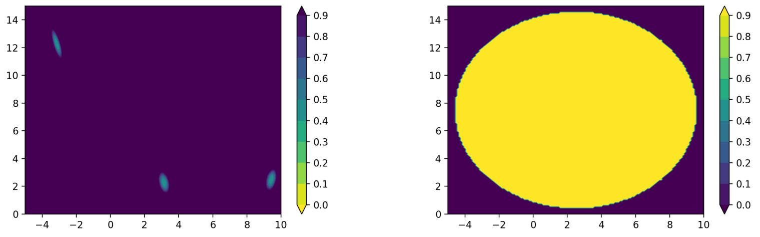

(a) Minima Locations (b) Disk Constraint

Figure 1: Constrained Bayesian optimization of the 2D Branin-Hoo Function.

The network has a single hidden layer with 50 hidden units, Gaussian activation functions and logistic output units. The mean parameters of q, the approximation to the true posterior, are initialized by sampling from a zero-mean Gaussian with variance $\frac{2}{d_{\text{in}}+d_{\text{out}}}$ according to the method of,[5] where d_{in} is the dimension of the previous layer in the network and d_{out} is the

dimension of the next layer in the network. The value of α is taken to be 0.5, minibatch sizes are taken to be 10 and 50 Monte Carlo samples are used to approximate the expectations with respect to q in each minibatch. The BNN adapted from[4] was implemented in the Theano library.[6] The LBFGs method[7] was used to optimize the EIC acquisition function in all experiments.

Results

The results of the sequential constrained Bayesian optimization algorithm with EIC are shown in Figure 2. The algorithm was initialized with 50 labeled data points drawn uniformly at random from the grid depicted. 40 iterations of Bayesian optimization were carried out.

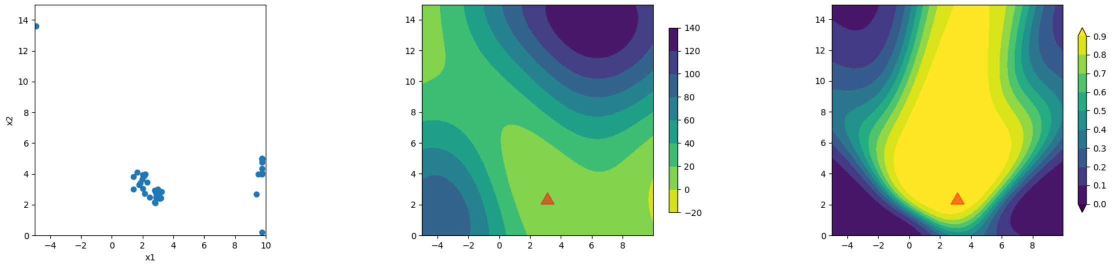

(a) Collected Data Points (b) Objective Predictive Mean (c) $\Pr(\mathcal{C}(\mathbf{x}) \geq 0)$

Figure 2: **a)** Data points collected over 40 iterations of sequential Bayesian optimization. **b)** Contour plot of the predictive mean of the sparse GP used to model the objective function. Lighter colours indicate lower values of the objective. **c)** The contour learned by the BNN giving the probability of constraint satisfaction.

The figures show that the algorithm is correctly managing to collect data in the vicinity of the single feasible minimum. Figure 3 compares the performance of parallel Bayesian optimization using the Kriging-Believer algorithm against the results of random sampling. Both algorithms were initialized using 10 data points drawn uniformly at random from the grid on which the Branin-Hoo function is defined and were run for 10 iterations of Bayesian optimization. At each iteration a batch of 5 data points was collected for evaluation.

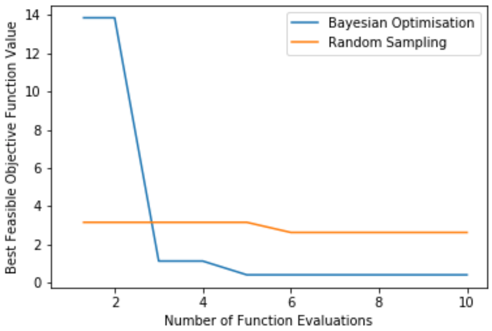

Figure 3: Performance of Parallel Bayesian Optimization with EIC against Random Sampling.

After 10 iterations, the minimum feasible value of the objective function was 0.42 for parallel Bayesian optimization with EIC using the Kriging-Believer algorithm and 2.63 for random sampling. The true minimum feasible value is 0.40.

Discussion

The Branin-Hoo experiment is designed to yield some visual intuition for the constrained Bayesian Optimization implementation in two dimensions before moving to higher dimensional molecular space. The results demonstrate that the implementation of constrained Bayesian optimization is behaving as expected in so far as the constraint in the problem is recognized and the search procedure outperforms random sampling.

It could be worth performing some investigation into how much worse the sparse GP performs relative to the exact GP in the constrained setting. Another aspect that could be explored is the impact of the initialization.

References

(1) Gelbart, M. A.; Snoek, J.; Adams, R. P. Bayesian optimization with unknown constraints. Proceedings of the Thirtieth Conference on Uncertainty in Artificial Intelligence. **2014**; pp 250–259.

(2) Bui, T. D.; Yan, J.; Turner, R. E. A Unifying Framework for Sparse Gaussian Process Approximation using Power Expectation Propagation. *arXiv preprint arXiv:1605.07066* **2016**,

(3) Kingma, D.; Ba, J. Adam: A method for stochastic optimization. *arXiv preprint arXiv:1412.6980* **2014**,

(4) Hernández-Lobato, J. M.; Li, Y.; Rowland, M.; Bui, T.; Hernández-Lobato, D.; Turner, R. E. Black-Box Alpha Divergence Minimization. Proceedings of The 33rd International Conference on Machine Learning. New York, New York, USA, 2016; pp 1511–1520.

(5) Glorot, X.; Bengio, Y. Understanding the difficulty of training deep feedforward neural networks. Proceedings of the Thirteenth International Conference on Artificial Intelligence and Statistics. Chia Laguna Resort, Sardinia, Italy, 2010; pp 249–256.

(6) Theano Development Team, Theano: A Python framework for fast computation of mathematical expressions. *arXiv e-prints* **2016**, *abs/1605.02688*.

(7) Liu, D. C.; Nocedal, J. On the limited memory BFGS method for large scale optimization. *Mathematical programming* **1989**, *45*, 503–528.
